# Deaths Associated with Respiratory Syncytial and Influenza Viruses among Persons ≥5 Years of Age in HIV-Prevalent Area, South Africa, 1998–2009^1^

**DOI:** 10.3201/eid2104.141033

**Published:** 2015-04

**Authors:** Stefano Tempia, Sibongile Walaza, Cecile Viboud, Adam L. Cohen, Shabir A. Madhi, Marietjie Venter, Claire von Mollendorf, Jocelyn Moyes, Johanna M. McAnerney, Cheryl Cohen

**Affiliations:** National Health Laboratory Service, Johannesburg, South Africa (S. Tempia, S. Walaza, S.A. Madhi, C. von Mollendorf, J. Moyes, J.M. McAnerney, C. Cohen);; Centers for Disease Control and Prevention, Atlanta, Georgia, USA (S. Tempia, A.L. Cohen);; Centers for Disease Control and Prevention, Pretoria, South Africa (S. Tempia, A.L. Cohen, M. Venter);; National Institutes of Health, Bethesda, Maryland, USA (C. Viboud);; University of the Witwatersrand, Johannesburg (S.A. Madhi, J. Moyes, C. Cohen)

**Keywords:** Influenza, viruses, respiratory syncytial virus, RSV, HIV, human immunodeficiency virus, mortality rates, deaths, South Africa

## Abstract

We estimated deaths attributable to influenza and respiratory syncytial virus (RSV) among persons >5 years of age in South Africa during 1998–2009 by applying regression models to monthly deaths and laboratory surveillance data. Rates were expressed per 100,000 person-years. The mean annual number of seasonal influenza–associated deaths was 9,093 (rate 21.6). Persons >65 years of age and HIV-positive persons accounted for 50% (n = 4,552) and 28% (n = 2,564) of overall seasonal influenza-associated deaths, respectively. In 2009, we estimated 4,113 (rate 9.2) influenza A(H1N1)pdm09–associated deaths. The mean of annual RSV-associated deaths during the study period was 511 (rate 1.2); no RSV-associated deaths were estimated in persons >45 years of age. Our findings support the recommendation for influenza vaccination of older persons and HIV-positive persons. Surveillance for RSV should be strengthened to clarify the public health implications and severity of illness associated with RSV infection in South Africa.

## Abstract 2

Mortality rates were higher among HIV-positive persons and older persons who had influenza.

Deaths Associated with Respiratory Viruses in HIV-Prevalent Area

Influenza virus and respiratory syncytial virus (RSV) infections cause substantial numbers of illness and deaths globally each year; the highest rates are in young children and persons >65 years of age (*1*–*4*). However, national estimates of deaths caused by these infections remain scarce in Africa.

Available data suggest that the severity of influenza and RSV illness is higher among HIV-positive persons (*5–11*). During 2009 in South Africa, ≈5.1 million HIV-positive persons >5 years of age were reported (*12*). The highest prevalence was in the 20–44-year age group, among whom HIV prevalence increased from 10% in 1998 to 24% in 2009. Coverage with highly active antiretroviral therapy (HAART) among HIV-positive persons slowly increased from 2004 to reach a plateau of ≈14% in 2009 (*12*). The lowest reported occurrence of HIV infection in the country was in persons >75 years of age; data showed a 0.6% prevalence and 26% HAART coverage for this group in 2009 (*12*). Pneumonia ranked within the top 5 leading causes of death among persons >15 years of age in South Africa in 2009 (*13*).

Clarification of the annual number of deaths associated with influenza and RSV in South Africa could assist with the prioritization of interventions. Because influenza virus and RSV infections are rarely confirmed by laboratory diagnosis and related deaths may be attributed to co-morbid conditions or secondary infections, we applied modeling approaches (*14*) to estimate seasonal and pandemic influenza- and RSV-associated deaths among persons >5 years of age during 1998–2009.

## Methods

### Mortality Data and Population Denominators

We obtained data on underlying causes of death for persons >5 years of age during 1998–2009 from Statistics South Africa (*15*). We used codes from the International Classification of Diseases, 10th Revision (ICD-10), to compile an age-specific (5–19, 20–44, 45–64, 64–74, and >75 years) monthly mortality data time series for all-cause (ICD-10: any); all respiratory (ICD-10: J00–J99); all circulatory (ICD-10: I00–I99); and pneumonia and influenza (P&I) (ICD-10: J10–J18), a subset of all respiratory deaths. During the study period, underreporting of deaths was estimated to be <5% (*13*). Population denominators were obtained from Statistics South Africa (*16*); we obtained year- and age-specific estimates of HIV prevalence in the population and HAART coverage among HIV-positive older children and adults from the Actuarial Society of South Africa AIDS and Demographic Model (*12*).

### Influenza and RSV Surveillance Data

For the study period before 2002, we obtained influenza virus data, including types and subtypes, from influenza-like illness surveillance implemented by the National Institute for Communicable Diseases, a division of the National Health Laboratory Service, South Africa (*17*), and RSV data from a cohort study (*10*). For the years after 2002, we acquired virologic data on influenza and RSV from the National Health Laboratory Services corporate data warehouse, a national database that includes all patients tested for respiratory viruses in the public sector in South Africa. We considered an influenza type or subtype to be dominant during the influenza season if it represented >50% of the circulating viruses.

### Estimation of Influenza- and RSV-Associated Deaths

We conducted a 2-stage analysis. In the first stage, we estimated the annual number of deaths associated with influenza and RSV in South Africa; in the second stage, we estimated the proportion of these deaths that were experienced by HIV-positive and HIV-negative persons. During the first stage, to estimate the number of deaths associated with seasonal and pandemic influenza and RSV, we fitted age-specific generalized regression models with a Poisson distribution and an identity link to the number of monthly deaths as previously described (*14*). The full model (model 1) included co-variates for time trends, seasonal variation, and proxies for viral circulation. Model specification, selection procedures and sensitivity analysis are provided in the Technical Appendix. Separate models were fitted for each age group and cause of death.

In South Africa, a diagnosis of AIDS is rarely coded on the death certificate (*13*), which hinders direct estimation of respiratory virus–associated deaths by HIV status. To assess the proportionate number of deaths associated with influenza and RSV among HIV-positive and HIV-negative persons, we used a previously developed methodology (*14*) that leverages the increasing trend in HIV prevalence in South Africa. The rationale is that if HIV is a risk factor for influenza- or RSV– associated death, influenza- or RSV– related mortality rates should increase over time proportionately to the observed increase in HIV prevalence. Our approach also controls for increasing HAART coverage, which may decrease the severity of influenza or RSV in HIV-positive patients (*18*). In this second-stage analysis, we fitted regression models (model 2) to annual estimates of influenza- and RSV-associated excess mortality rates by age group, including data for HIV prevalence and HAART coverage, time trends, and dominant influenza subtypes (for the influenza model) (*14*). Model specifications are provided in the Technical Appendix.

Subsequently, we obtained mortality rates associated with HIV status by dividing the estimated influenza-associated deaths by HIV status from model 2 by the mid-year population estimates within each category (i.e., HIV-positive and HIV-negative persons). Mortality rates were expressed per 100,000 person-years. We used log-binomial regression (*14*) to estimate age-specific and age-adjusted relative risk for influenza- and RSV-associated death related to HIV infection by comparing influenza- or RSV-associated mortality rates among HIV-positive and HIV-negative persons. We used STATA version 12 (StataCorp, College Station, Texas, USA) to implement the statistical analysis.

### Sensitivity Analysis of Influenza- and RSV-Associated Deaths among Persons >45 Years of Age

In contrast to previous studies (*3*,*4*), our models estimated that influenza, but not RSV, was associated with excess deaths in South Africa among persons aged >45 years. We hypothesized that differences in the timing of the RSV and influenza seasons may explain these discrepancies because RSV and influenza rarely co-circulate in South Africa, but they do in other temperate settings (*3*,*4*). This difference could potentially confound burden of illness estimates for these pathogens. We implemented a sensitivity analysis to explore this hypothesis by artificially shifting the RSV surveillance time series so that it overlapped with the influenza season and repeating model 1 calculations (Technical Appendix).

### Ethics

This analysis used only publicly available mortality data and deidentified and aggregated laboratory data. Therefore, the study was considered to be exempt from human subjects ethics review.

## Results

### Deaths and Mortality Rates

South Africa had a population of ≈44.8 million persons >5 years of age in 2009; persons 5–64 years of age accounted for 85% of this population. During the study period, a mean of 463,594 deaths occurred annually among persons from South Africa >5 years of age, of which 101,450 (22%) were attributable to respiratory and 112,716 (24%) to circulatory causes of death (Technical Appendix Table 1). The mean annual mortality rate for all-cause death increased from 112 for persons 5–19 years of age to 9,732 for persons >75 years of age. Similar patterns were observed regarding the other underlying causes of death evaluated in this study. Among persons 20–44 years of age, for which the HIV burden is greatest (24% HIV prevalence in 2009 [*12*]), the annual mortality rate for all respiratory deaths increased from 78 in 1998 to 310 in 2004, subsequently declining to 233 in 2009 (monthly trends provided in the Figure, panel A). In contrast, no evident secular trend for all respiratory mortality rates was observed among persons >75 years of age, for whom HIV incidence is lowest (<0.01% HIV prevalence in 2009 [*12*]) (Figure, panel B).

**Figure:**
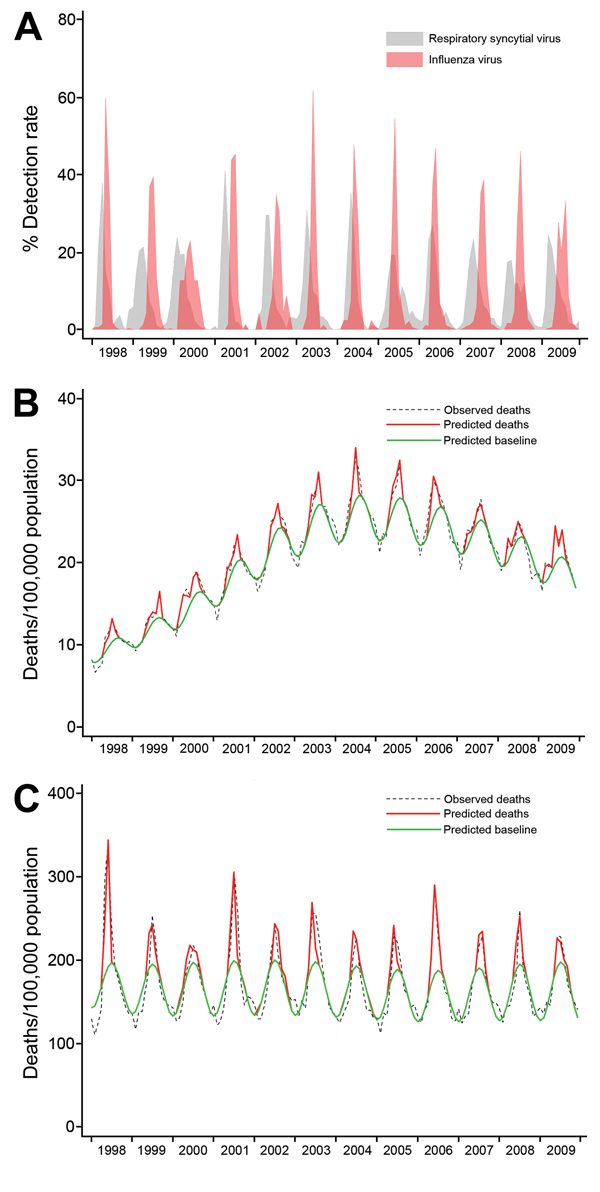
Monthly mortality and detection rates for influenza and respiratory syncytial virus in South Africa, 1998–2009. A) Observed respiratory deaths, predicted deaths, and predicted baseline by month (model 1) of persons 20–44 years of age. B) Observed respiratory deaths, predicted deaths, and predicted baseline by month (model 1) in persons ≥75 years of age. C) Detection rate (i.e., monthly number of positive specimens divided by annual number of specimens tested) of influenza and respiratory syncytial virus (all ages).

### Influenza and RSV Laboratory Surveillance

A mean of 3,403 (range 227–15,321) and 1,810 (range 578–5,247) respiratory specimens were tested annually for influenza virus and RSV, respectively. The mean annual number of specimens that tested positive was 937 (27%) for influenza virus and 356 (20%) for RSV. During the study period, the influenza season peaked between May and August (winter in South Africa); 10 of the 12 years showed peak activity during June–July (Figure, panel C). In 2009, an epidemic of influenza A(H3N2) peaked in June, and influenza A(H1N1)pdm09 activity peaked in August. RSV peak activity was observed during March and April (autumn in South Africa) in 8 of the 12 years. Early or late peaks were observed in February or May in the remaining years.

### Influenza- and RSV-Associated Deaths

During 1998–2009, the estimated annual number of all-cause seasonal influenza-associated deaths in persons >5 years of age (model 1) ranged from 6,450 to 11,012 (rate 16.7–24.5) (Technical Appendix Table 2). In the same population, estimated annual all-cause RSV-associated deaths ranged from 292 to 626 (rate 0.7–1.4) (Technical Appendix Table 2).

The estimated mean seasonal influenza–associated mortality rate for all-cause deaths increased progressively from 0.8 for the 5–19-year age group to 379.2 for the >75-year age group. Similar trends were observed for the other causes of death evaluated in this study (Table 1). Overall, the estimated mean seasonal influenza–associated mortality rate for all-cause deaths was higher for HIV-positive persons than for those who were HIV-negative (age-adjusted relative risk [aRR] 7.9, 95% CI 7.1–8.9). Overall, 28% (2,564/9,093) of estimated all-cause seasonal influenza–associated deaths occurred among HIV-positive persons.

**Table 1:** Seasonal influenza virus mean annual excess deaths and relative risk for death related to HIV infection among persons ≥5 y of age, South Africa, 1998–2009*

Cause of death by age, y	Mean annual excess deaths (95% CI)									Relative risk, HIV+ vs HIV– (95% CI)
	Total				HIV+			HIV–		
	No., mean	Rate,† mean	% Death over model baseline		No., mean	Rate,† mean		No., mean	Rate,† mean	
All causes										
5–19	127 (91–171)	0.8 (0.6–1.1)	2.4 (2.0–2.7)		52 (37–70)	23.6 (16.9–31.8)		75 (53–101)	0.5 (0.3–0.7)	46.6 (20.9–104.3)
20–44	1,966 (1,160–2,770)	10.7 (6.3–15.1)	3.0 (2.9–3.2)		1,851 (1,074–2,567)	56.3 (33.1–78.2)		114 (86–203)	0.7 (0.6–1.3)	72.8 (38.1–138.9)
45–64	2,447 (1,499–3,408)	37.3 (23.0–52.0)	5.7 (5.5–5.8)		661 (400–920)	163.9 (101.6–228.2)		1,785 (1,098–2,488)	29.3 (18.1–40.9)	5.6 (5.0–6.2)
65–74	1,664 (1,185–2,181)	115.4 (82.5–151.3)	8.1 (7.8–8.6)		NED	NED		1,664 (1,185–2,181)	115.4 (82.5–151.3)	NA
≥75	2,888 (2,138–3,557)	379.2 (281.1–467.1)	10.7 (10.2–11.7)		NED	NED		2,888 (2,138–3,557)	379.2 (281.1–467.1)	NA
≥5	9,093 (6,073–12,087)	21.6 (15.7–28.9)	5.7 (5.5–6.1)		2,564 (1,511–3,557)	64.7 (38.3–81.1)		7,189 (4,560–8,530)	18.9 (12.5–24.3)	7.9 (7.1–8.9)‡
All respiratory										
5–19	96 (60–137)	0.6 (0.4–0.9)	9.8 (8.9–10.2)		55 (24–79)	24.6 (15.3–32.7)		41 (27–60)	0.3 (0.2–0.4)	87.7 (30.0–157.3)
20–44	778 (416–1,144)	4.2 (2.3–6.2)	5.0 (4.7–5.2)		722 (386–1061)	21.5 (11.6–31.7)		56 (30–82)	0.4 (0.2–0.6)	57.1 (22.5–143.4)
45–64	1,106 (696–1,562)	16.8 (10.6–23.8)	11.4 (11.0–11.7)		380 (237–536)	93.9 (59.9–132.7)		725 (459–1,025)	11.9 (7.6–16.8)	7.9 (6.6–9.4)
65–74	626 (416–852)	43.4 (28.9–59.1)	14.3 (13.7–15.1)		NED	NED		626 (416–852)	43.4 (28.9–59.1)	NA
≥75	1,005 (704–1,323)	132.3 (92.9–174.2)	17.3 (16.8–21.4)		NED	NED		1,005 (704–1,323)	132.3 (92.9–174.2)	NA
≥5	3,613 (2,292–5,018)	8.5 (5.8–11.2)	10.0 (9.6–10.8)		1157 (647–1,676)	28.7 (17.1–38.8)		2,455 (1,636–3,342)	6.4 (4.8–8.2)	11.1 (9.4–13.1)‡
All circulatory										
5–19	28 (7–48)	0.2 (0.05–0.3)	6.2 (5.8–6.9)		NED	NED		28 (7–48)	0.2 (0.05–0.3)	NA
20–44	252 (127–375)	1.4 (0.7–2.0)	4.0 (3.5–4.7)		226 (120–332)	7.2 (3.8–10.6)		26 (14–36)	0.2 (0.1–0.3)	41.2 (15.3–123.7)
45–64	854 (619–1,081)	13.2 (9.6–16.7)	6.9 (5.9–7.3)		258 (116–400)	66.3 (29.8–102.8)		596 (322–870)	9.9 (5.3–14.4)	6.6 (3.3–9.9)
65–74	749 (536–955)	52.1 (37.3–66.4)	8.3 (7.8–8.9)		NED	NED		749 (536–955)	52.1 (37.3–66.4)	NA
≥75	1,270 (971–1,511)	167.4 (128.2–199.2)	10.1 (9.3–11.1)		NED	NED		1,270 (971–1,511)	167.4 (128.2–199.2)	NA
≥5	3,153 (2,260–3,970)	7.5 (5.8–12.3)	7.8 (7.1–8.7)		484 (236–732)	12.0 (5.8–18.2)		2,669 (1,859–3,420)	6.9 (4.8–8.9)	6.8 (3.1–10.5)‡
Pneumonia and influenza										
5–19	86 (55–120)	0.6 (0.4–0.8)	13.7 (11.9–15.6)		50 (31–69)	22.4 (14.4–31.3)		36 (23–51)	0.2 (0.1–0.3)	91.1 (28.9–186.7)
20–44	569 (317–823)	3.1 (1.7–4.5)	5.4 (4.9–6.1)		522 (290–754)	15.5 (8.7–22.5)		47 (26–68)	0.3 (0.2–0.4)	48.5 (17.6–133.1)
45–64	612 (378–923)	9.3 (5.7–13.9)	12.5 (11.1–13.4)		279 (169–415)	67.1 (42.0–101.1)		336 (209–507)	5.5 (3.4–8.3)	12.2 (9.4–15.6)
65–74	299 (179–430)	20.8 (12.5–29.8)	16.1 (15.3–17.5)		NED	NED		299 (179–430)	20.8 (12.5–29.8)	NA
≥75	620 (438–870)	83.0 (57.8–114.6)	21.2 (19.4–24.6)		NED	NED		620 (438–870)	83.0 (57.8–114.6)	NA
≥5	2,186 (1,367–3,166)	5.2 (2.4–6.1)	10.8 (8.7–12.2)		848 (490–1,238)	20.9 (13.8–28.1)		1,341 (875–1,926)	3.5 (2.1–5.7)	17.3 (13.6–21.8)‡

*Estimated from model 1 (excess deaths irrespective of HIV status) and model 2 (excess deaths by HIV status). HIV, human immunodeficiency virus; NED, no estimated deaths; NA, not applicable.

†Mortality rates per 100,000 person-years.

‡Age-adjusted relative risk.

In 2009, we estimated 4,113 (rate 9.2) all-cause influenza A(H1N1)pdm09–associated deaths among persons >5 years of age (Table 2). The mortality rate associated with influenza A(H1N1)pdm09 in 2009 in persons 5–19 years of age was 5.4 times higher than the mean for prepandemic years. In contrast, persons >75 years of age experienced ≈100 times lower influenza A(H1N1)pdm09 rates than expected in typical nonpandemic years. A similar trend was observed for other causes of death evaluated in this study.

**Table 2:** Influenza A(H1N1)pdm09 excess deaths among persons ≥5 y of age, South Africa, July–September 2009*

Cause of death by age, y	Influenza A(H1N1)pdm09 excess deaths in 2009 (95% CI)			Mortality rate ratio† (95% CI)
	No.	Rate†	% Death over model baseline (95% CI)	
All causes				
5–19	682 (455–910)	4.4 (2.9–5.9)	12.7 (8.5–17.0)	5.4 (4.5–6.6)
20–44	1820 (1201–2403)	9.3 (6.2–12.4)	2.6 (1.7–3.5)	0.9 (0.6–1.2)
45–64	1301 (867–1735)	17.2 (11.5–23.0)	2.5 (1.7–3.4)	0.5 (0.3–0.7)
65–74	279 (186–373)	17.6 (11.8–23.5)	1.2 (0.8–1.6)	0.2 (0.1–0.3)
≥75	31 (20–42)	3.6 (2.4–4.8)	0.1 (0.06–1.3)	0.01 (0.006–0.013)
≥5	4113 (2729–5463)	9.2 (6.1–12.2)	2.6 (1.9–3.3)	0.4 (0.3–0.5)§
All respiratory				
5–19	626 (417–835)	4.1 (2.7–5.4)	61.6 (41.1–82.2)	6.9 (5.5–8.6)
20–44	936 (624–1248)	4.8 (3.2–6.4)	6.0 (4.0–8.0)	1.2 (1.1–1.3)
45–64	729 (486–972)	9.6 (6.4–12.8)	6.3 (4.2–8.4)	0.6 (0.5–0.7)
65–74	159 (106–212)	10.1 (6.7–13.4)	3.3 (2.2–4.4)	0.2 (0.1–0.3)
≥75	16 (10–21)	1.8 (1.2–2.5)	0.2 (0.1–0.3)	0.01 (0.008–0.022)
≥5	2466 (1,643–3,288)	5.5 (3.6–7.3)	7.1 (5.5–8.7)	0.7 (0.6–0.8)§
All circulatory				
5–19	7 (0–13)	0.05 (0.00–0.1)	1.7 (0.0–3.3)	0.2 (0.1–0.6)
20–44	252 (168–336)	1.3 (0.8–1.7)	4.1 (2.7–5.5)	1.0 (0.8–1.2)
45–64	404 (269–539)	5.3 (3.5–7.1)	3.0 (2.0–4.0)	0.4 (0.3–0.5)
65–74	75 (51–103)	4.7 (3.2–6.3)	0.8 (0.5–1.1)	0.1 (0.07–0.12)
≥75	13 (8–17)	1.5 (1.0–2.0)	0.09 (0.06–0.12)	0.01 (0.004–0.014)
≥5	751 (496–1008)	1.7 (1.1–2.2)	1.7 (1.3–2.0)	0.2 (0.1–0.3)§
Pneumonia and influenza				
5–19	449 (297–586)	2.9 (1.9–3.9)	73.1 (48.7–97.4)	5.5 (4.3–6.9)
20–44	548 (365–731)	2.8 (1.8–3.7)	5.7 (3.8–7.6)	0.9 (0.7–1.1)
45–64	421 (281–562)	5.6 (3.7–7.4)	7.3 (4.8–9.7)	0.6 (0.5–0.7)
65–74	90 (54–126)	5.7 (2.8–5.9)	4.3 (2.9–5.8)	0.3 (0.2–0.4)
≥75	3 (0–12)	0.4 (0.0–1.9)	0.08 (0.0–0.2)	0.005 (0.001–0.009)
≥5	1511 (997–2017)	3.4 (2.2–4.5)	3.7 (2.9–4.5)	0.7 (0.6–0.8)§

*Estimated from model 1 (excess deaths irrespective of HIV status).

†Mortality rates per 100,000 person-years

‡Mortality rate ratio: 2009 influenza A(H1N1)pdm09 vs. 1998–2009 mean annual seasonal influenza.

§Age-adjusted rate ratio.

The estimated RSV-associated mortality rate for all-cause deaths was 0.4 for the 5–19-year age group and 2.4 for the 20–44-year age group (Table 3). However, no RSV-associated deaths were estimated among persons >45 years of age. Among persons 5–44 years of age, the RSV-associated mortality rate for all causes of death was considerably higher for HIV-positive than HIV-negative persons (aRR 66.1, 95% CI 26.0–167.8). Similar trends were observed for the RSV-associated mortality rate among all respiratory and P&I deaths. A nonsignificant RSV-associated mortality rate (mean annual deaths: 8) was identified among circulatory deaths only in the 5–19-year age group. Overall, 89% (455/511) of all-cause RSV-associated death occurred among HIV-positive persons.

**Table 3:** Respiratory syncytial virus mean annual excess deaths and relative risk for death related to HIV infection among persons ≥5 y of age, South Africa, 1998–2009*

Cause of death by age, y	Mean annual excess deaths (95%CI)									Relative risk, HIV+ vs. HIV– (95% CI)
	Total				HIV+			HIV–		
	No., mean	Rate,† mean	% Death over model baseline		No., mean	Rate,† mean		No., mean	Rate,† mean	
All causes										
5–19	61 (29–87)	0.4 (0.2–0.6)	0.9 (0.8–1.1)		25 (12–36)	11.6 (5.6–16.5)		36 (17–51)	0.2 (0.1–0.3)	58.4 (15.1–154.9)
20–44	449 (66–863)	2.4 (0.4–4.7)	0.6 (0.5–0.7)		430 (62–818)	13.1 (2.1–24.9)		19 (4–45)	0.1 (0.02–0.30)	98.3 (20.6–163.2)
45–64	NED	NED	NED		NED	NED		NED	NED	NA
65–74	NED	NED	NED		NED	NED		NED	NED	NA
≥75	NED	NED	NED		NED	NED		NED	NED	NA
≥5	511 (95–950)	1.2 (0.4–1.5)	0.7 (0.6–0.8)		455 (74–854)	12.1 (5.3–19.4)		55 (21–96)	0.1 (0.04–0.25)	66.1 (26.0–167.8)‡
All respiratory										
5–19	39 (13–61)	0.3 (0.1–0.4)	3.6 (3.3–3.8)		22 (9–33)	10.1 (4.2–14.2)		16 (7–24)	0.1 (0.03–0.18)	90.2 (16.5–193.2)
20–44	389 (269–516)	2.1 (1.7–2.5)	2.3 (2.1–2.4)		369 (255–489)	11.1 (8.3–14.8)		20 (13–26)	0.1 (0.07–0.14)	81.7 (17.3–182.5)
45–64	NED	NED	NED		NED	NED		NED	NED	NA
65–74	NED	NED	NED		NED	NED		NED	NED	NA
≥75	NED	NED	NED		NED	NED		NED	NED	NA
≥5	429 (186–573)	1.0 (0.04–1.2)	1.1 (0.8–1.3)		392 (264–522)	9.8 (6.3–13.1)		37 (20–50)	0.1 (0.04–0.12)	85.4 (27.2–167.8)‡
All circulatory										
5–19	8 (0–30)	0.05 (0–0.2)	1.4 (0.8–1.7)		NED	NED		8 (0–30)	0.05 (0–0.2)	NA
20–44	NED	NED	NED		NED	NED		NED	NED	NA
45–64	NED	NED	NED		NED	NED		NED	NED	NA
65–74	NED	NED	NED		NED	NED		NED	NED	NA
≥75	NED	NED	NED		NED	NED		NED	NED	NA
≥5	8 (0–30)	0.05 (0–0.2)	1.4 (0.8–1.7)		NED	NED		8 (0–30)	0.05 (0–0.2)	NA
Pneumonia and influenza										
5–19	35 (16–52)	0.2 (0.1–0.3)	5.2 (4.5–6.2)		20 (9–30)	9.2 (4.2–13.5)		15 (6–21)	0.1 (0.05–0.14)	92.2 (15.3–159.7)
20–44	257 (178–340)	1.4 (0.7–2.2)	2.3 (1.5–2.8)		243 (168–321)	7.2 (4.7–11.1)		14 (10–19)	0.1 (0.6–1.3)	73.4 (11.9–145.9)
45–64	NED	NED	NED		NED	NED		NED	NED	NA
65–74	NED	NED	NED		NED	NED		NED	NED	NA
≥75	NED	NED	NED		NED	NED		NED	NED	NA
≥5	292 (194–392)	0.7 (0.4–1.0)	1.3 (0.9–1.5)		263 (177–351)	6.6 (5.1–7.2)		29 (16–40)	0.1 (0.6–1.4)	82.7 (23.1–196.6)‡

*Estimated from model 1 (excess deaths irrespective of HIV status) and model 2 (excess deaths by HIV status). HIV, human immunodeficiency virus; NED, no estimated deaths; NA, not applicable.

†Mortality rates per 100,000 person-years.

‡Age-adjusted relative risk.

### Sensitivity Analysis of Influenza- and RSV-Associated Deaths among Persons >45 Years of Age

On sensitivity analysis, we applied an artificial incremental shift of the RSV laboratory surveillance time series to make the influenza and RSV seasons more synchronous, resulting in a progressive increase in estimated RSV-associated deaths (Technical Appendix Table 3). We found that annual all-cause RSV-associated deaths peaked at 3,661 among persons >45 years of age (compared with 0 in the main analysis) when the RSV and influenza seasons coincided in most years (2 months’ incremental shift of the RSV season). Thereafter, the estimated mean annual all-cause RSV-associated deaths decreased again to 0 when the peak circulation of the 2 pathogens were farther apart (5-month incremental shift of the RSV season). This trend was observed for all causes of death evaluated in this study. Conversely, the estimated influenza-associated deaths remained stable throughout sensitivity analyses, without regard to the shift in RSV season. Specifically, the estimated all-cause influenza-associated deaths among persons >45 years of age remained within 10% of its original value (Technical Appendix Table 3).

## Discussion

We reported estimates of influenza- and RSV-associated deaths in persons >5 years of age in a high HIV prevalence setting in Africa. The number of seasonal influenza–associated deaths was substantial and observed across age groups and underlying causes of death evaluated in this study, irrespective of the person’s HIV status. However, the seasonal influenza-associated mortality rates were highest among persons >75 years of age and HIV-positive adults 5–64 years of age. The seasonal influenza–associated deaths in these groups accounted for 50% and 28%, respectively, of the total influenza-associated deaths among persons >5 years of age. Conversely, a moderate number of deaths associated with RSV infection was found mainly among HIV-positive persons 5–44 years of age; the model did not estimate RSV-associated deaths for persons >45 years of age.

Previous studies have reported elevated influenza-associated mortality rates among the elderly (*3,4,19–21*) and HIV-positive persons (*6,8,9,11,14*). We did not find an excess risk for seasonal influenza–associated death due to HIV infection among persons >75 years of age across the underlying causes of deaths evaluated in this study. This finding may reflect the low HIV prevalence among elderly persons that may have hindered our ability to estimate the extent of disease in this group using our described method.

Among persons >5 years of age years in South Africa, the number of deaths associated with pandemic influenza A(H1N1)pdm09 during 2009 was approximately half that of an average influenza season in prepandemic years. However, pandemic-related mortality rates were higher in the 5–19-year age group and lower in the >45-year age group compared with typical seasons. Other studies have reported overall lower mortality rates associated with the first year of circulation of the 2009 pandemic virus, compared with that of seasonal influenza, but have found a higher disease burden for children and young adults (*4,21–25*). Our estimates are similar to the lower-bound estimates for South Africa from a global influenza A(H1N1)pdm09 mortality model (*26*).

For persons >5 years of age in South Africa, ≈90% of RSV-associated deaths were estimated to have occurred among HIV-positive persons 5–44 years of age, although our model did not estimate RSV-associated deaths among persons >45 years of age, an age group in which the HIV infection rate is low (1.3% in 2009) (*12*). This finding suggests that HIV infection may be a major risk factor for RSV-associated death for persons >5 years of age, consistent with our high estimate of HIV as a risk factor for RSV-associated death (aRR 66.1, 95% CI 26.0–167.8). Other studies have reported an increased risk for RSV-associated death among HIV-positive persons (*5,7*).

Our findings differ from those of similar studies from the United States and England, where RSV-associated deaths have been reported across age groups (including persons referred to as elderly in the respective studies) and where the influenza and RSV seasons are, in most cases, synchronous (*3,4*). However, there are notable geographic variations in the timing of RSV circulation across the United States (*27*). In southern Florida, where the RSV season precedes the influenza season by several weeks as in South Africa, 1 peak of pneumonia hospitalizations among persons >65 years of age could be detected concurrently with the influenza season. In contrast, among children <5 years of age, 2 distinct peaks of pneumonia hospitalizations were observed concomitantly with the RSV and influenza seasons (*27*). Further, a study that used a methodology similar to ours and evaluated data from a large hospital group in South Africa estimated elevated RSV-associated hospitalizations among children <5 years of age but no RSV-associated hospitalizations among adult and elderly persons (*28*). These results are similar to a previous study conducted in South Africa that found influenza- and RSV-associated mortality among children <5 years of age (*14*), but no RSV-associated mortality was estimated among elderly persons in our study.

We performed a sensitivity analysis to test whether the overlap between the RSV and influenza seasons affected mortality estimates. We found that RSV mortality estimates increased substantially among persons >45 years of age when the RSV season was artificially shifted to later in the year, so that it coincided with influenza activity. However, influenza estimates remained within 10% of their main analysis values, solidifying our influenza results. This finding suggests that careful interpretation of the results of time series excess mortality models is needed when used to simultaneously estimate the mortality attributable to co-circulating pathogens, particularly for RSV.

Severe illness and death among laboratory-confirmed RSV-infected adults has been reported (*29,30*), even though the clinical association between pathogen detection and disease remains difficult to interpret in the absence of comparison groups (i.e., RSV prevalence among adults without respiratory illness). Studies conducted in Kenya and South Africa that compared the RSV prevalence among patients hospitalized with severe acute respiratory illness (SARI) to control groups found that RSV infection was associated with hospitalization among children <5 years of age, but no association was found among persons >5 years of age (*31,32*). This finding may suggest that, although RSV is detected among older children and adults, it may play a less important role as a pathogen in this group. However, both studies were underpowered to look specifically at disease association in persons >65 years of age. Studies conducted in Egypt, Guatemala, Kenya, and Thailand, where patients of all ages hospitalized with acute lower respiratory tract infections were systematically enrolled and tested using PCR techniques, reported RSV detection rates of <1%–5% among persons >50 or >75 years of age, compared with RSV detection rates of >20% in infants and young children (*31,33–35*). Reinfection with RSV during life has been reported (*36*), but titers of serum-neutralizing antibodies >6 (log2 scale) have been associated with 3 times lower risk for RSV-associated hospitalizations (*37*). Adults reinfected with RSV may have high levels of serum-neutralizing antibodies that have potential to lower the prevalence and severity of RSV-associated hospitalizations in this age group. 

In South Africa during 2009–2010, the RSV detection rate among patients hospitalized with SARI decreased from 26.8% among infants <1 year of age to 0.9% among persons >75 years of age. In the same study population, the influenza detection rate across age groups was 8%–12% (*38*). The low RSV detection rate among adults and elderly persons with SARI suggests a lower rate of RSV-associated hospitalization than that for influenza for this group (and as a result, a potentially low number of RSV-associated deaths).

Although RSV-associated deaths among persons >45 years of age are expected to occur in South Africa, our modeling approach may fail to statistically estimate a small number of cases. Ecologic studies conducted in settings similar to ours, where influenza and RSV peak activities are not synchronous, may assist in better differentiating the relative impact of these pathogens, especially in adults. In addition, results obtained from ecologic models should be interpreted along with findings from case-based studies and the strengths and weaknesses of both approaches should be evaluated.

Our study has limitations that warrant discussion. First, the lack of weekly mortality statistics and the paucity of virologic data before 2002 may have hindered the ability to accurately estimate the relative contribution of RSV and influenza virus on number of associated deaths. Second, the lack of influenza incidence data (such as influenza-like illness indicators) hampered our ability to consider more refined indicators of respiratory virus activity in our time series models as reported by Goldstein et al. in 2012 (*39*). Third, because of poor records of HIV infection in the death register documenting the early years of our study, we used indirect methods to estimate the number of deaths associated with respiratory viruses among HIV-positive and HIV-negative persons. Although the HIV epidemic in South Africa is considered to be a major factor responsible for the increased mortality rates observed over the years (*40*), the lack of time series data for other potential co-occurring conditions and risk factors may have resulted in overestimation of the increased risk for death associated with HIV infection. Last, we could not estimate the influenza A(H1N1)pdm09–associated mortality by HIV status because our method requires availability of HIV prevalence data over several years of A(H1N1)pdm09 circulation.

In conclusion, we report a substantial risk for death associated with seasonal influenza virus infection, especially for persons >75 years of age and HIV-positive adults 20–64 years of age. The risk for death associated with RSV was mainly found among HIV-positive persons 5–44 years of age; our model did not identify excess RSV-associated deaths in persons >45 years of age. We also report low to moderate numbers of RSV-associated deaths among persons >5 years of age; however, clinical diagnosis and surveillance for RSV should be continued and strengthened to better describe the consequences and severity associated with RSV infection in this age group.

Technical Appendix Model selection procedures, mortality data and estimates of influenza- and RSV-associated deaths by year and from sensitivity analysis.
