# Supplementary material for: Deaths Associated with Respiratory Syncytial and Influenza Viruses among Persons ≥5 Years of Age in HIV-Prevalent Area, South Africa, 1998–20091
Source: Emerg Infect Dis. 2015 Apr;21(4):600–8. doi: 10.3201/eid2104.141033 (PMC4378466; doi:10.3201/eid2104.141033)
Supplement: Technical Appendix — Model selection procedures, mortality data and estimates of influenza- and RSV-associated deaths by year and from sensitivity analysis. [file 14-1033-Techapp-s1.pdf]

Article DOI: http://dx.doi.org/10.3201/eid2104.141033

Deaths Associated with Respiratory Syncytial and Influenza Viruses among Persons ≥5 Years of Age in HIV-Prevalent Area, South Africa, 1998–2009

Technical Appendix

Methods

Stage 1 Model: National Estimates of Influenza- and RSV-associated Deaths

To estimate the influenza- (seasonal and pandemic) and RSV-associated deaths, we fitted age-specific Generalized Linear Models (GLM) with a Poisson distribution and an identity link to the number of monthly deaths as previously described (*1*). The identity link was selected because it is considered the most biologically plausible link to model the impact of pathogen circulation on mortality (*2*–*6*).The full model (Model 1) included covariates for time trends and seasonal variation as well as proxies for viral circulation as follows:

$$E(Y_{i,t}) = \beta_{0,\mathrm{i}} + \beta_{1,i}[t] + \beta_{2,i}[t^2] + \beta_{3,i}[t^3] + \beta_{4,i}[t^4] + \beta_{5,i}[\sin(2t\pi/12)] + \beta_{6,i}[\cos(2t\pi/12)]$$

$$+\beta_{7,i}[Seasonal\_Influenza(t)] + \beta_{8,i}[\mathrm{A}(H1N1)\ pdm09] + \beta 9,i\ [RSV(t)] + \varepsilon_{i,t}$$

$E(Y_{i,t})$ represents age-specific number of deaths in age group i and month t; $\beta_{0,i}$ is the age-specific model constant; $\beta_{1,i}$ to $\beta_{4,i}$ are age-specific coefficients associated with time trends (linear to quartic polynomial terms); $\beta_{5,i}$ and $\beta_{6,i}$ are age-specific coefficients associated with harmonic terms accounting for annual background seasonal variations; $\beta_{7,i}$ to $\beta_{9,i}$ are age-specific coefficients representing the contribution of respiratory viruses to mortality (seasonal influenza ($\beta_{7,i}$): including A(H1N1), A(H3N2) and B; pandemic influenza ($\beta_{8,i}$): A(H1N1)pdm09; and RSV ($\beta_{9,i}$)); and $\varepsilon_{i,t}$ is the age-specific error term. *Seasonal_influenza(t)*, *A(H1N1)pdm09(t)* and *RSV(t)* are proxies for monthly viral activity, estimated as the monthly number of specimen testing positive for influenza or RSV over the annual number of specimens tested for the specific

pathogen. We used standardization by the annual total of all specimens tested for the specific pathogen, to reduce possible bias associated with differences in specimen sampling and laboratory methods over time (*7*). Proxies for viral activity were based on all-age laboratory surveillance data and hence remained the same across all age groups. Models were fitted separately for each age group and cause of death.

Through model selection procedures, we assessed the fit of models including higher order polynomials to represent more subtle time trends (1st to 6th degree) and additional harmonic terms representing annual and semi-annual periodicity ($sin(2t_i\pi/12)$ and $cos(2t_i\pi/12)$; $sin(4t_i\pi/12)$ and $cos(4t_i\pi/12)$). The final model (Model 1) was that for which the Akaike value (AIC) was minimized, that is, the model that provided best fit to the data while maintaining parsimony. We also assessed the effect of removing the RSV covariate on the model estimates for influenza virus. In this model the estimates for influenza-associated deaths remained within 10% of their main-analysis values, solidifying our influenza results. Furthermore, we assessed the model fit using two different proxies for monthly viral activities. Proxy 1 was obtained by dividing the monthly number of specimen testing positive for influenza or RSV to the annual number of specimens tested for the specific pathogen (model presented in equation 1). Proxy 2 was not standardized by the annual number of specimens tested. The estimates from models using proxies 1 or 2 were comparable; however the models fitted using proxy 2 yielded consistently higher AIC values compared to the models fitted using proxy 1. In addition, we implemented a sensitivity analysis where we compared the estimates from our count model (equation 1) to those obtained from a rate model. The estimates obtained from the rate models remained within 3% of their main-analysis values (count model) across the analysis implemented over the different causes of death and age groups evaluated in the study (Technical Appendix Table 4). Modeling counts or rates in equation 1 gives similar results given slow trends in population sizes. Given the minimal difference between the estimates from the count and rates models, we chose to model death counts instead of death rates in line with previously developed methodologies (*1, 8,9*). We also considered b-splines (1 knot per month was best) instead of polynomial terms to model background seasonality, but polynomial terms provided the best fit to the South African data, perhaps because of the relatively crude monthly resolution of the data; however this hypothesis could not be tested.

We estimated the age-specific excess deaths associated with influenza and RSV each month by subtracting an expected baseline from the monthly deaths predictions of Model 1. The baseline was obtained by setting the relevant viral covariates to 0 (i.e., to obtain a baseline for seasonal influenza, we set the seasonal influenza proxy to 0). Annual excess deaths were estimated as the sum of the monthly excess deaths for each year. We obtained the 95% confidence interval (CI) for the estimated excess deaths using bootstrap resampling on blocks of calendar years (12 months block resampling with replacement) over 1000 replications (*1,10*). For each resampled dataset we refitted the regression model and the 95% CI were obtained from the 2.5th and 97.5th percentiles of the estimated influenza- and RSV-associated deaths from the 1000 resampled datasets.

Stage-2 Model: Estimates of Influenza- and RSV-associated Deaths by HIV Status

In South Africa, a diagnosis of AIDS is rarely coded on the death certificate (*11*) hindering direct estimation of respiratory virus-associated excess deaths by HIV status. Instead, we used a two-stage regression approach that builds on the annual national excess deaths estimates provided by Model 1. The rationale for the annual regression relies on using the increasing trend in HIV prevalence over time to estimate the fraction of national excess deaths attributed to HIV-positive and negative persons (in particular, if HIV was not a risk factor for influenza-related death, then influenza-related mortality rates would not increase over time, and influenza-related deaths would occur among HIV-infected and HIV-uninfected populations proportionally to their group sizes). The annual regression further accounts for increasing HAART coverage (which tends to decrease influenza-related excess deaths among HIV-infected persons over time [*9*]) and circulation of more severe influenza subtypes (A(H3N2) vs A(H1N1) or B). Through model selection procedures, we assessed the fit of models including higher order polynomials (1st to 3rd degree) to represent time trends of health indicators unrelated to influenza, HIV or HAART as well as the removal of the influenza, HIV or HAART covariate from the models. The model with 2nd degree polynomial time trends and influenza, HIV and HAART covariates had the best fit to the data. The model accounts for the combined effect of varying HIV prevalence in the population over the years as well as different HIV interventions, including the prevention of HIV infection and the effect of HAART on HIV infected persons.

We fitted separate multivariate GLM for each age group and cause of death, considering a Poisson distribution and an identity link (Model 2) as previously described (1). The following model was used for influenza:

$$E\left(Y_{i,t}\right) = \alpha_i \begin{pmatrix} \beta_{0,i} + \beta_{1,i}[t] + \beta_{2,i}[t^2] + \beta_{3,i}[Influenza\_Subtype\ (t)] \\ + \beta_{4,i}[HIV_i(t)] + \beta_{5,i}[HAART_i(t)] + \varepsilon_i(t) \end{pmatrix}$$

Where $E(Y_{i,t})$ represents the age-specific number of influenza-associated excess deaths in age group i and year t (as obtained from the stage-1 approach); α_i is an offset representing the population size of age group i; $\beta_{0,i}$ is the age-specific intercept; $\beta_{1,i}$ and $\beta_{2,i}$ are age-specific coefficients associated with time trends (linear and quadratic) included to account for potential variations of health indicators unrelated to influenza, HIV prevalence or HAART coverage in the population; $\beta_{3,i}$ is the age-specific coefficient associated with dominant seasonal influenza type/subtype each year (categorical variable with A(H3N2)-dominant years as reference group versus A(H1N1) or B) (*7,12*); $\beta_{4,i}$ is the coefficient associated with the HIV prevalence in the population in age group i and year t; $\beta_{5,i}$ is the coefficient associated with HAART coverage among HIV-infected persons in the population in age group i and year t; and ε_i is the age-specific error term. The model was not fitted to death estimates for influenza A(H1N1)pdm09 as the approach requires several years of circulation of a specific virus to partition excess deaths by HIV status.

Similar models, with the exclusion of the dominant influenza types/subtypes, were used to estimate RSV-associated mortality rates by HIV status. We estimated the influenza and RSV-related excess deaths among HIV-infected patients by subtracting an expected baseline from the Model 2 annual estimates. The baseline was obtained by setting the HIV and HAART covariates to 0.

Sensitivity Analysis of Influenza- and RSV-associated Deaths among Persons ≥45 Years of Age

In South Africa the RSV season typically precedes the influenza season by several weeks (*13–15*) while in other countries such as the United States of America and England, the influenza and RSV seasons are, in most cases, more synchronous (*12,16*). In these settings, both influenza- and RSV-associated deaths have been reported across age groups including elderly persons (*12,16*), while in our study we estimated only influenza-associated deaths among persons aged ≥45 years. To determine if synchronous RSV and influenza seasons may confound excess

death estimates derived from time series regression models, we implemented a sensitivity analysis whereby we applied a 1 month incremental shift (1 to 5 months) of the RSV laboratory proxy indicator while keeping the influenza proxy as in the main analysis. We then refitted the monthly regression model of stage 1 (equation 1) for each underlying cause of death evaluated in this study among persons ≥45 years of age. Hence, we obtained estimates of influenza- and RSV-associated deaths under each scenario whereby the RSV season progressively approached and then diverged from the influenza season. This allowed us to model artificially synchronous RSV and influenza seasons and compare our estimates with those of the asynchronous seasons observed in South Africa.

Results

Technical Appendix Table 1. Deaths among persons ≥5 y of age, South Africa, 1998–2009

	Deaths	
Cause of death, age, y	No., mean (range)	Rate,* mean (range)
All causes		
5–19	16,974 (13,300–19,282)	112 (92–126)
20–44	190,910 (108,052–233,926)	1,045 (653–1,257)
45–64	124,156 (87,355–148,521)	1,899 (1,596–2,090)
65–74	57,660 (50,294–64,230)	4,009 (3,871–4,146)
≥75	73,893 (61,318–88,604)	9,732 (9,122–10,277)
≥5	463,594 (321,917–541,780)	1,101 (837–1,250)
All respiratory		
5–19	2,887 (1,800–3,496)	19 (12–23)
20–44	44,054 (19,247–57,459)	240 (116–310)
45–64	27,206 (1,7492–33,170)	414 (319–474)
65–74	11,987 (10,558–13,156)	834 (801–870)
≥75	15,314 (13,353–17,619)	2,024 (1,882–2,189)
≥5	101,450 (63,971–122,326)	240 (166–282)
All circulatory		
5–19	1,371 (1,262–1,567)	9 (8–10)
20–44	17,681 (15,515–19,435)	97 (91–107)
45–64	34,799 (30,285–38,674)	537 (506–572)
65–74	24,890 (23,003–26,751)	1,735 (1,658–1,828)
≥75	33,975 (30,171–39,207)	4,487 (4,240–4,735)
≥5	112,716 (100,327–123,683)	269 (256–283)
Pneumonia and influenza		
5–19	1,874 (1,152–2,281)	12 (8–15)
20–44	29,615 (11,827–38,562)	162 (71–212)
45–64	13,571 (7,364–17,335)	206 (134–249)
65–74	4,973 (4,252–5,472)	346 (327–368)
≥75	7865 (6,749–9,020)	1,040 (959–1,149)
≥5	57,901 (32,332–71,860)	137 (84–165)

*Mortality rate per 100,000 person-years.

Technical Appendix Table 2. Seasonal influenza- and respiratory syncytial virus-associated deaths among persons ≥5 y of age categorized by HIV status, South Africa, 1998–2009*

Year, predominant influenza type (subtype)		Excess all causes deaths						Excess all respiratory deaths					
		Total		HIV+		HIV–		Total		HIV+		HIV–	
		No.	Rate†	No.	Rate†	No.	Rate†	No.	Rate†	No.	Rate†	No.	Rate†
Influenza viruses													
1998, A(H3N2)		8,859	21.5	2,552	68.1	6,901	18.4	3,601	8.7	1,181	31.5	2,420	6.4
1999, B		6,833	17.4	1,411	60.7	5,674	15.4	2,576	6.5	538	23.1	2,038	5.5
2000, A(H1N1)		7,292	18.3	1,707	53.2	5,927	16.1	2,823	7.0	700	21.8	2,122	5.7
2001, A(H3N2)		8,020	19.8	2,098	63.0	6,380	17.1	3,191	7.8	922	27.7	2,269	6.1
2002, B		6,450	16.7	1,158	64.5	5,472	14.9	2,351	6.1	400	31.5	1,950	5.3
2003, A(H3N2)		9,310	22.2	2,855	69.8	7,164	19.0	3,823	9.1	1,356	33.1	2,466	6.5
2004, A(H3N2)		9,784	23.1	3,120	71.4	7,486	19.7	4,021	9.4	1,495	34.2	2,526	6.6
2005, A(H1N1)		10,015	23.3	3,229	70.4	7,678	18.3	4,087	9.5	1,537	33.5	2,549	6.6
2006, A(H3N2)		10,229	23.6	3,255	68.4	7,904	19.4	4,130	9.5	1,528	32.1	2,601	6.7
2007, A(H3N2)		10,565	24.1	3,240	66.1	8,276	19.2	4,210	9.6	1,491	30.4	2,719	6.9
2008, A(H1N1)		10,746	22.2	3,143	62.5	8,531	19.7	4,241	9.5	1,419	28.2	2,821	6.1
2009, A(H3N2)		11,012	24.5	3,006	58.5	8,878	22.4	4,302	9.6	1,324	25.7	2,978	7.5
Respiratory syncytial virus													
1998	NA	292	0.7	237	13.2	55	0.1	183	0.4	154	8.5	29	0.08
1999	NA	323	0.8	273	11.7	50	0.1	224	0.5	194	8.3	29	0.08
2000	NA	363	0.9	316	11.8	47	0.1	274	0.6	245	7.6	29	0.08
2001	NA	436	1.1	384	11.5	51	0.1	358	0.8	323	9.7	35	0.08
2002	NA	514	1.2	458	12.2	55	0.1	449	1.0	410	10.9	39	0.08
2003	NA	574	1.3	516	12.6	58	0.1	518	1.2	476	11.6	42	0.08
2004	NA	615	1.4	554	12.7	60	0.1	559	1.3	516	11.8	43	0.08
2005	NA	626	1.4	565	12.3	60	0.1	561	1.3	519	11.3	42	0.08
2006	NA	621	1.4	562	11.8	59	0.1	547	1.2	506	10.6	41	0.08
2007	NA	608	1.3	551	11.2	57	0.1	521	1.1	482	9.8	42	0.08
2008	NA	589	1.3	534	10.6	55	0.1	491	1.1	454	9.0	43	0.08
2009	NA	569	1.2	515	10.0	54	0.1	461	1.0	426	8.2	42	0.08

* Estimated from stage 1 model (excess deaths irrespective of HIV status) and stage 2 model (excess deaths by HIV status).
†Mortality rates per 100,000 person-years

Technical Appendix Table 3. Sensitivity analysis of seasonal influenza- and respiratory syncytial virus-associated mortality implemented over one month incremental shift of the respiratory syncytial virus season among persons ≥45 y of age in South Africa, 1998–2009

Shift of RSV* season (in months)	Mean annual excess deaths							
	All causes		All respiratory		All circulatory		Pneumonia and influenza	
	Influenza	RSV	Influenza	RSV	Influenza	RSV	Influenza	RSV
+0†	6,442	0	2,523	0	2,647	0	1,450	0
+1	6,478	1,709	2,609	209	2,730	470	1,499	149
+2	5,811	3,661	2,309	1,329	2,406	1,598	1,244	1,089
+3	6,500	2,668	2,548	1,136	2,686	1,473	1,444	903
+4	6,635	0	2,652	167	2,796	107	1,539	201
+5	6,103	0	2,504	0	2,553	0	1,451	0

*RSV, respiratory syncytial virus.
†Actual data with no shift of the RSV season.

Technical Appendix Table 4. Sensitivity analysis comparing the results of seasonal and pandemic influenza- and respiratory syncytial virus-associated deaths between the count model (main model; equation 1) and the rate model, South Africa, 1998–2009*

	Mean annual excess number of deaths					
	Seasonal influenza, mean 1998–2009		Influenza A(H1N1)pdm09, 2009		Respiratory syncytial virus, mean 1998–2009	
Cause of death, age, yr	Count model (95% CI)	Rate model	Count model (95% CI)	Rate model	Count model (95% CI)	Rate model
All causes						
5–19	127 (91–171)	131	682 (455–910)	699	61 (29–87)	62
20–44	1,966 (1,160–2,770)	1,926	1,820 (1,201–2,403)	1,801	449 (66–863)	445
45–64	2,447 (1,499–3,408)	2,434	1,301 (867–1,735)	1,317	NED	NED
65–74	1,664 (1,185–2,181)	1,680	279 (186–373)	285	NED	NED
≥75	2,888 (2,138–3,557)	2,879	31 (20–42)	32	NED	NED
All respiratory						
5–19	96 (60–137)	95	626 (417–835)	615	39 (13–61)	38
20–44	778 (416–1,144)	791	936 (624–1,248)	927	389 (269–516)	386
45–64	1,106 (696–1,562)	1,088	729 (486–972)	734	NED	NED
65–74	626 (416–852)	629	159 (106–212)	161	NED	NED
≥75	1,005 (704–1,323)	1,012	16 (10–21)	16	NED	NED
All circulatory						
5–19	28 (7–48)	27	7 (0–13)	7	8 (0–30)	8
20–44	252 (127–375)	254	252 (168–336)	257	NED	NED
45–64	854 (619–1,081)	860	404 (269–539)	399	NED	NED
65–74	749 (536–955)	738	75 (51–103)	74	NED	NED
≥75	1,270 (971–1,511)	1,289	13 (8–17)	13	NED	NED
Pneumonia and influenza						
5–19	86 (55–120)	88	449 (297–586)	444	35 (16–52)	36
20–44	569 (317–823)	565	548 (365–731)	560	257 (178–340)	252
45–64	612 (378–923)	626	421 (281–562)	416	NED	NED
65–74	299 (179–430)	307	90 (54–126)	91	NED	NED
≥75	620 (438–870)	605	3 (0–12)	3	NED	NED

*NED, no estimated deaths.

References

1. Tempia S, Walaza S, Viboud C, Cohen AL, Madhi SA, Venter M, et al. Mortality associated with seasonal and pandemic influenza and respiratory syncytial virus among children <5 years of age in a high HIV prevalence setting—South Africa, 1998–2009. Clin Infect Dis. 2014;58:1241–9. PubMed http://dx.doi.org/10.1093/cid/ciu095

2. Gay NJ, Andrews NJ, Trotter CL, Edmunds WJ. Estimating deaths due to influenza and respiratory syncytial virus—reply. JAMA. 2003;289:2499; author reply 2500–2. PubMed http://dx.doi.org/10.1001/jama.289.19.2499-a

3. Simonsen L, Blackwelder WC, Reichert TA, Miller MA. Estimating deaths due to influenza and respiratory syncytial virus. JAMA. 2003;289:2499–500, author reply 2500–2. PubMed http://dx.doi.org/10.1001/jama.289.19.2499-b

4. Glezen WP, Couch RB. Estimating deaths due to influenza and respiratory syncytial virus. JAMA. 2003;289:2500; author reply 2500–2. PubMed http://dx.doi.org/10.1001/jama.289.19.2500-a

5. Goldstein E, Viboud C, Charu V, Lipsitch M. Improving the estimation of influenza-related mortality over a seasonal baseline. Epidemiology. 2012;23:829–38. PubMed http://dx.doi.org/10.1097/EDE.0b013e31826c2dda

6. Boshuizen HC, Feskens EJ. Fitting additive Poisson models. Epidemiol Perspect Innov. 2010;7:4 PubMed http://dx.doi.org/10.1186/1742-5573-7-4

7. Zhou H, Thompson WW, Viboud CG, Ringholz CM, Cheng PY, Steiner C, et al. Hospitalization associated with influenza and respiratory syncytial virus in the United States, 1993–2008. Clin Infect Dis. 2012;54:1427–36. PubMed http://dx.doi.org/10.1093/cid/cis211

8. Cohen C, Simonsen L, Sample J, Kang JW, Miller M, Madhi SA, et al. Influenza-related mortality among adults aged 25–54 years with AIDS in South Africa and the United States of America. Clin Infect Dis. 2012;55:996–1003. PubMed http://dx.doi.org/10.1093/cid/cis549

9. Dushoff J, Ploykin JB, Viboud C, Earn DJ, Simonsen L. Mortality due to influenza in the United States – an annualized regression approach using multiple-cause mortality data. Am J Epidemiol. 2005;163:181–7. PubMed http://dx.doi.org/10.1093/aje/kwj024

10. Weinberger DM, Simonsen L, Jordan R, Steiner C, Miller M, Viboud C. Impact of the 2009 influenza pandemic in pneumococcal pneumonia hospitalizations in the United States. J Infect Dis. 2012;205:458–65. PubMed http://dx.doi.org/10.1093/infdis/jir749

11. Statistics South Africa. Mortality and cause of death in South Africa, 1999 through 2009. Pretoria, Statistics South Africa, 2009.

12. Thompson WW, Shay DK, Weintraub E, Brammer L, Cox N, Anderson LJ, et al. Mortality associated with influenza and respiratory syncytial virus in the United States. JAMA. 2003;289:179–86. PubMed http://dx.doi.org/10.1001/jama.289.2.179

13. Moyes J, Cohen C, Pretorius M, Groome M, von Gottberg A, Wolter N, et al. Epidemiology of respiratory syncytial virus-associated acute lower respiratory tract infection hospitalizations among HIV-infected and HIV-uninfected South African children, 2010–2011. J Infect Dis. 2013;208(Suppl 3):S217–26. PubMed http://dx.doi.org/10.1093/infdis/jit479

14. Pretorius MA, Madhi SA, Cohen C, Naidoo D, Groome M, Moyes J, et al. Respiratory viral co-infections identified by a 10-plex real-time reverse-transcription polymerase chain reaction assay in patients hospitalized with severe acute respiratory illness—South Africa, 2009–2010. J Infect Dis. 2012;206(Suppl 1):S159–65. PubMed http://dx.doi.org/10.1093/infdis/jis538

15. Tempia S, Walaza S, Viboud C, Cohen AL, Madhi SA, Venter M, et al. Mortality associated with seasonal and pandemic influenza and respiratory syncytial virus among children <5 years of age in a high HIV prevalence setting – South Africa, 1998–2009. Clin Infect Dis. 2014;58:1241–9. PubMed http://dx.doi.org/10.1093/cid/ciu095

16. Hardelid P, Pebody R, Adrews N. Mortality caused by influenza and respiratory syncytial virus by age group in England and Wales 1999–2010. Influenza Other Respir Viruses. 2013;7:35–45. PubMed http://dx.doi.org/10.1111/j.1750-2659.2012.00345.x
